# Characterization of Cytomegalovirus Disease in Solid Organ Transplant Recipients by Markers of Inflammation in Plasma

**DOI:** 10.1371/journal.pone.0060767

**Published:** 2013-04-08

**Authors:** Halvor Rollag, Thor Ueland, Anders Åsberg, Anders Hartmann, Alan G. Jardine, Atul Humar, Mark D. Pescovitz, Angelo A. Bignamini, Pål Aukrust

**Affiliations:** 1 Department of Microbiology, Oslo University Hospital, Rikshospitalet, Oslo, Norway; 2 Research Institute of Internal Medicine, Oslo University Hospital, Rikshospitalet, Oslo, Norway; 3 Department of Pharmaceutical Biosciences, School of Pharmacy, University of Oslo, Oslo, Norway; 4 Department of Organ Transplantation, Gastroenterology and Nephrology, Section for Nephrology, Oslo University Hospital, Rikshospitalet, Oslo, Norway; 5 Section of Clinical Immunology and Infectious Diseases, Oslo University Hospital, Rikshospitalet, Oslo, Norway; 6 Faculty of Medicine, University of Oslo, Oslo, Norway; 7 Department of Medicine, University of Glasgow, Glasgow, United Kingdom; 8 Department of Medicine, University of Alberta, Edmonton, Canada; 9 Departments of Surgery and Microbiology/Immunology, Indiana University, Indianapolis, Indiana, United States of America; 10 School of Specialization in Hospital Pharmacy, University of Milan, Milan, Italy; University of Birmingham, United Kingdom

## Abstract

**Background:**

While several studies have examined the general inflammatory responses in relation to cytomegalovirus infection, the identification of the various inflammatory mediators as well as their relative importance is far from clear.

**Patients and Methods:**

Solid organ recipients enrolled in an international multicenter trial of cytomegalovirus disease treatment (the VICTOR study) were analyzed (n = 289) (ClinicalTrials.gov NCT00431353). Plasma markers of inflammation and endothelial cell activation were assessed at baseline by enzyme immunoassays.

**Results:**

The major findings were: (i) Plasma levels of the CXC-chemokine interferon-inducible protein-10 (P<0.001) and C-reactive protein (P = 0.046) were independently associated with the presence of cytomegalovirus DNAemia above lower level of quantification. (ii) High levels of CC-chemokine ligand 21 (P = 0.027) and pentraxin 3 (P = 0.033) were independently associated with tissue invasive cytomegalovirus disease as opposed to cytomegalovirus syndrome.

**Conclusion:**

Our findings illustrate the complex interaction between cytomegalovirus and the immune system, involving a wide range of inflammatory mediators that could be associated to disease manifestations in cytomegalovirus related disease.

## Introduction

Cytomegalovirus (CMV) is a major viral pathogen affecting solid organ transplant recipients, and is responsible for substantial morbidity in affected individuals. The clinical manifestations of CMV include an acute viral syndrome as well as tissue invasive diseases including pneumonitis, hepatitis and gastrointestinal disease [1]. In addition to these CMV-induced manifestations, CMV has immunomodulatory effects that predispose to opportunistic infections as well as acute and chronic allograft rejection in the infected host [1], [2].

Cytokines and chemokines orchestrate the interaction between CMV and the immune system, as they participate in reactivation of latent virus and regulation of viral replication [3]. In addition to host-encoded cytokines and chemokines, the virus itself encodes homologues of these proteins and their receptors as an immune escape strategy [4], [5]. The CMV-mediated inflammatory responses are also important determinants of tissue damage and clinical manifestations of CMV infection [6]. However, while several studies have examined the general inflammatory responses in relation to CMV infection, the identification of the specific inflammatory mediators and their relative importance in relation to pathogenesis of CMV disease is unclear.

We have recently reported that pretreatment levels of inflammatory markers were associated with virological and clinical outcomes in solid organ transplant recipients with CMV infection [7]. Herein we in the same study population investigated (i) if the inflammatory markers differed between patients with CMV DNAemia as compared with those with CMV-DNA levels below the detection limit of the assay, but with similar clinical symptoms and (ii) the association between inflammatory markers and clearly defined tissue invasive CMV disease as opposed to CMV syndrome. The inflammatory markers that were analyzed included general downstream markers of inflammation (C-reactive protein [CRP] and pentraxine-3 [PTX3]), markers of upstream inflammatory pathways (soluble tumor necrosis factor receptor type 1 [sTNF-R1]), a marker of endothelial cell activation (von Willebrand factor [vWF]) and certain relevant chemokines (*r*egulated upon *a*ctivation, *n*ormal *T* cell *e*xpressed and *s*ecreted [RANTES]/CCL5, CC-chemokine ligand (CCL16), CCL21 and interferon-γ-inducible protein 10 [IP-10]/CXCL10), and (i) the presence or absence of confirmed CMV DNAemia by the central laboratory [2], and in those with verified CMV infection, (ii) the presence of CMV disease manifestations.

## Materials and Methods

### Study Design

The present *post-hoc* analyses were performed on plasma samples obtained at baseline in the VICTOR trial. This was an international multicenter trial of oral valganciclovir versus intravenous ganciclovir for the treatment of CMV disease in adult solid organ transplant recipients (ClinicalTrials. gov NCT00431353) [2]. Between April 2004 and June 2006, 321 patients were included in the main study. The present post-hoc study includes available baseline samples from the pooled patient population (both treatment arms) that were suspected to have CMV disease by local assessments, in total 289 samples. At the central laboratory were 235 of these patients verified to be CMV DNAemia-positive (18300 CMV-DNA copies/mL −3420−765000 [median, 25%−75% percentile]) and 54 were shown to have CMV-DNAemia below the lower level of quantification (LLoQ) (<600 CMV-DNA-copies/mL) which is the limit at which an assay reliably reports results (Table 1). Detailed inclusion and exclusion criteria are presented elsewhere [2]. For the analyses of inflammatory markers in relation to manifestation of CMV disease, adult solid organ transplant recipients with both virological and clinical evidence of CMV disease (regardless of donor or recipient CMV serostatus) were eligible for enrollment (n = 235). Patients who were considered to have life-threatening CMV disease by the investigator were excluded [2]. Immunosuppressive therapy followed local practice.

**Table 1:** Demographic data of patients suspected to have CMV disease and confirmed or not confirmed by a central analyses of CMV DNAemia.

		DNAemia positive (n = 235)	DNAemia negative (n = 54)	P-value
**Sex**	Male (%)	145 (61.7)	33 (61.1)	0.936[a]
**Age**	Years (mean±SD)	45.1±13.7	42.4±11.9	0.171[b]
**Weight**	Kg (mean±SD)	67.5±15.9	67.7±14.0	0.952[b]
**HLA AB-DR mismatch**	N (%)Missing	152 (94.1) 74	26. (96.1) 27	0.686[a]
**CMV D/R serotypes at** **time of Tx**	D+/R D+/R+ D−/R+ D−/R- Missing	45 (19.1) 91 (38.7) 15(6.3) 13 (5.5) 71	3 (7.9) 28 (51.8)4 (7.1) 3 (5.7) 16	0.080[a]
**Time after Tx**	Days; median [range-interquartile]	69 [48–133]	159 [92–1178]	<0.001[c]
**Transplanted organ**	Kidney; N (%) Liver; N (%) Heart;N (%) Lung; N (%)	180 (76.6) 11 (9.4) 18(7.7) 15 (6.4)	48 (88.9) 3 (5.6)1 (1.9) 2 (3.7)	0.225[a]
**Clinical diagnosis**	Syndrome; N (%) TI disease; N (%)	135 (57.4) 100 (42.6)	28 (51.9) 26 (48.1)	0.455[a]
**Ethnicity**	Caucasian; N (%) Black; N (%) Oriental;N (%) Hispanic; N (%) Other; N (%)	190 (80.9) 5 (2.1) 25(10.6) 8 (3.4) 7 (3.0)	34 (63.0) 3 (5.6)5 (9.3) 9 (16.7) 3 (5.6)	0.001[a]

[a] chi square.

[b] t-test.

[c] Mann-Whitney test.

### Ethics Statement

The clinical trial was conducted in accordance with the Declaration of Helsinki, good clinical practice guidelines and applicable local regulatory requirements. Both the “Victor study” (S-04011) and the present *post-hoc* analysis (SPREK 2010/3464) were approved by the Regional Committee for Medical Research Ethics, South East Norway Section B. Informed written consent was obtained from all patients included in this analysis.

### Definition of CMV Disease

The definitions are outlined in detail in previous publications and were in accordance with published guidelines at the time of the study [8]–[10]. In short, CMV disease was defined as the presence of CMV DNAemia in plasma above LLoQ in addition to clinical signs of disease. For viral syndrome at least one of the following additional features was required: body temperature ≥38°C, new or increased significant malaise, leucopenia (white blood cell count of <3,500/µl), atypical lymphocytosis of ≥5%, or thrombocytopenia (platelet count of <100,000/µl). Tissue invasive disease was defined based on evidence of localized CMV infection (CMV inclusion cells or *in situ* detection of CMV antigen or DNA by immunization or hybridization, respectively) in a biopsy or other appropriate specimen (e.g., bronchoalveolar lavage [BAL] and cerebrospinal fluid [CSF]) and/or relevant symptoms or signs of organ dysfunction that was unlikely to be due to other causes after relevant tests to exclude such causes had been performed.

### Virological Laboratory Analyses

Plasma CMV DNAemia was assessed using the Amplicor CMV Monitor® Test (Roche Diagnostics, Indianapolis, IN) applying a LLoQ of 600 copies/mL plasma. Measurement of CMV IgG levels was determined in plasma using the Architect CMV-IgG kit® (Abbott Diagnostic Division, Abbott Park, IL). LLoQ was 6 U/mL and the upper limit was 250 U/mL. The determination of human herpes virus-6 (HHV-6), HHV-7 and Epstein Barr virus (EBV) viral loads, using quantitative real-time PCR on whole blood samples was performed as previously described [11], [12]. All analyses were performed at central laboratories.

### Cytokine Assays

Markers of inflammation were analyzed in EDTA plasma, collected and stored as previously described [7], by enzyme immunoassays (EIA) obtained from R&D Systems (Stillwater, MN) except for vWF which was analyzed by EIA with antibodies from Dako Cytomatio (Glostrup, Denmark) [13]. The assays had been validated and kit independent controls were run on every test plate. Based on data from our laboratory, the inter- (n = 5) and intra-assay (n = 10) variation coefficients of the assays were: high-sensitivity CRP (0.9%, 7.1%), PTX3 (8.0%, 5.2%), sTNF-R1 (6.4%, 1.6%), RANTES (9.9%, 4.7%), CCL21 (8.5%, 16.7%), CXCL16 (3.3%, 4.4%) and IP-10 (5.2%, 7.2%) and vWF (<10.0%, <10.0%).

### Statistical Methods

Data were analyzed without replacement of missing data. Demographics were compared with the usual t-test, chi square and Mann-Whitney procedures. The differences in cytokine levels between tested groups were examined with the analysis of variance (ANOVA), adjusted for sex, age (<45/≥45 years), time from transplant (<90/90–180/>180 days) and baseline viral load (<10,000/10,000–50,000/>50,000 copies/ml). This last adjustment was not applied when testing the presence of CMV (since it would result in co-linearity with the factor being tested). To monitor potential associations of cytokines with clinical conditions, we ran a multivariable logistic regression using the stepwise backward elimination procedure, starting with all the mentioned variables and all cytokine values and using P<0.10 to eliminate variables from the model. For each variable found to be independently associated with outcome, we assigned integer weights, taking into account also the cut-off values of the Receiver Operating Characteristic (ROC) curves, to each category of that variable to produce an overall score. The weights corresponded approximately to the relative magnitude of the coefficient for the category in the multivariable model. A weight of 0 was assigned to the category with the lowest event prevalence for each variable. The integer weights for each variable were summed to obtain the total score for each patient. The score was then tested with the ROC analysis, tested for the difference from randomness (AUC = 0.500) under the non-parametric assumption. All statistical analyses were performed with SPSS Statistics 17.0 for Windows (IBM Corp., Somers, NY). Throughout, we report two-tailed p values and values <0.05 for multivariate analyses, or <0.00625 for univariate analysis (to account for multiplicity, according to the conservative Bonferroni criterion, as the 8 tested markers should be considered correlated) were considered significant.

## Results

### Plasma Levels of Inflammation Markers in Relation to the Presence of CMV DNAemia

**Figure 1:**
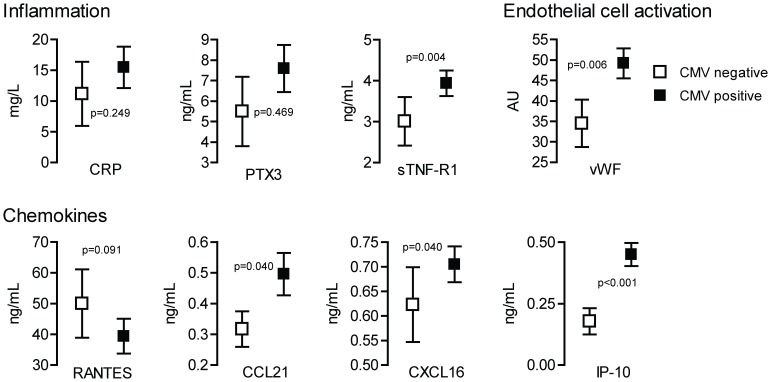
Markers of inflammation in relation to CMV-DNAemia. Plasma levels of markers of inflammation, marker of endothelial cell activation and chemokines in relation to presence or absence of CMV DNAemia in patients suspected to have CMV disease. The P-values are adjusted for potential confounders (i.e., age, sex and time from transplantation). P-values <0.00625 were considered significant to account for multiple testing (see Methods). Data are given as mean and 95% CI.

Fifty-four of the patients with suspected CMV infection had CMV DNA in plasma below LLoQ. We were therefore able to compare CMV-DNAemia below (n = 54) and CMV-DNAemia above LLoQ (n = 235) in patients with comparable clinical symptoms. As shown in Figure 1, patients with detectable CMV DNAemia had significantly higher plasma levels of sTNF-R1 (P = 0.004), vWF (P = 0.006), and IP-10 (P = 0.001), whereas a non-significant trend for higher plasma levels of CCL21 (P = 0.040) and CXCL16 (P = 0.040) was observed, as compared with patients with CMV-DNAemia below LLoQ.

When performing ROC analyses, vWF, CRP, IP-10, CCL21 and sTNF-R1 were found to be associated with detectable CMV DNA in plasma as opposed to patients with similar symptoms and CMV DNAemia below LLoQ. Once included together with potential confounders (age, sex and time from transplant) in the multivariable logistic regression IP-10 (OR [per each 10 units increase]: 1.498; 95% CI: 1.221–1.839; P<0.001), CRP (OR: 1.002; 95%CI: 1.000–1.004; P = 0.046), and time from transplant (OR: 0.449; 95% CI: 0.290–0.696; P = 0.001) were confirmed to be independently associated with positive CMV DNAemia.

### Plasma Levels of Inflammatory Markers in Relation to Clinical Manifestation of CMV Disease, Univariate Analyses

**Figure 2:**
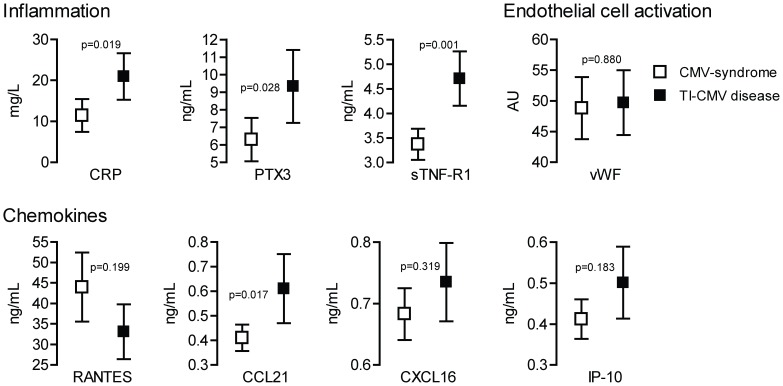
Markers of inflammation in relation to CMV-disease. Plasma levels of markers of inflammation, marker of endothelial cell activation and chemokines in relation to clinical manifestation of CMV disease as either CMV syndrome or tissue invasive CMV disease. The P-values are adjusted for potential confounders (i.e., age, sex, viral load and time from transplantation). TI, tissue invasive. P-values <0.00625 were considered significant to account for multiple testing (see Methods). Data are given as mean and 95% CI.

Those patients characterized to have tissue invasive CMV disease had markedly higher levels of the inflammatory marker sTNF-R1 (0.001) and a non-significant trend for CRP (P = 0.019), PTX3 (P = 0.028) and the homeostatic chemokine CCL21 (P = 0.017) compared with those with CMV syndrome (Figure 2). In contrast to the association with CMV DNAemia, vWF and IP-10 levels were not related to the clinical manifestation of CMV disease (Figure 2); nor were viral load associated with tissue invasive CMV disease (P = 0.054).

### Plasma Levels of Inflammatory Markers in Relation to Clinical Manifestation of CMV Disease, Multivariate Analyses

Based on ROC analyses, CRP, CCL21, PTX3 and sTNF-R1 were found to be potential “predictors” of tissue invasive disease. Once used with potential confounders (age, sex, time from transplant and baseline viral load) in the multivariable logistic regression, CCL21 (OR per each ng/mL: 3.865; 95% CI: 1.167–12.807; P = 0.027), PTX3 (OR per each 10 ng/mL: 1.473; 95% CI: 1.033–2.102; P = 0.033) and time from transplant (OR: 1.952; 95% CI: 1.295–2.942; P = 0.009) were confirmed to be independently associated with tissue invasive CMV disease.

From the logistic coefficients and the cut-off values of the ROC curves, a score was constructed (Table 2). Using this composite score, a fair ROC curve is obtained, with a reasonable diagnostic value; AUC 0.702 (95% CI: 0.634 to 0.770; P<0.001). Patients with a score ≤4 have an odds-ratio of 4.69 (2.63–8.36; P<0.001) of having CMV syndrome, rather than tissue invasive disease, in comparison with those having a score of 4 or more. The score had sensitivity: 0.757 (95% CI: 0.661–0.838); specificity: 0.600 (95% CI: 0.51–0.685); diagnostic accuracy: 0.668 (95% CI: 0.603–0.729); positive predictive value: 0.591 (95% CI: 0.500–0.677); negative predictive value: 0.765 (95% CI: 0.670–0.843). In broad terms, in addition to time since transplantation (>90 days), high levels of PTX3 and CCL21 were associated with tissue invasive CMV disease.

**Table 2:** Composite score constructed from the multivariable logistic coefficients and ROC curve cut-offs of predictors of tissue invasive CMV disease and corresponding diagnostic characteristics.

Score	0	1	2	3	4
**Time from transplantation (days)**	<90		90–180		>180
**PTX3 (ng/mL)**	≤2.5	>2.5–8.0		>8.0	
**CCL21 (ng/mL)**	≤0.25		>0.25–0.65	>0.65	

If ≥4 points: OR 4.7 (95% CI: 2.6–8.4) for having tissue invasive CMV disease.

### Plasma Levels of Inflammatory Markers in Relation to Type of Transplanted Organ

The adjusted ANOVA analysis evidenced only two significant differences associated with the type of transplanted organs. Compared with all other patients, lung-transplant recipients had higher CRP (+158%, P = 0.025) and lower sTNF-R1 (−30%, P = 0.003). However, the lung transplant recipients represented the smallest group (n = 15) and yielded the most heterogeneous values. The differences may thus be explained by chance and unbalanced data distribution rather than true differences.

### Plasma Levels of Inflammatory Markers in Relation to Other Variables

In contrast to the association with CMV DNAemia and disease manifestation, the inflammatory markers showed no association with CMV-IgG serostatus, HLA-AB/DR mismatches or viral co-infections by HHV-6, HHV-7, EBV or any combination thereof (data not shown).

## Discussion

In a previous study we showed that plasma levels of CXCL16, PTX3 and vWF at start of treatment were independently associated with virological and clinical treatment failure during anti-CMV-therapy in solid organ recipients [7]. In the present study we advance these findings by showing (i) an association between certain inflammatory markers and the presence of CMV DNAemia as opposed to solid organ transplant recipients with similar symptoms but with no CMV DNAemia, (ii) an association between a certain “cytokine signature” and well-defined invasive CMV diseases as opposed to CMV syndrome in these patients. Thus, while CRP and IP-10 were independently associated with the presence of CMV DNAemia above LLoQ, high levels of PTX3 and CCL21 were independently associated with tissue invasive CMV disease. Our findings further underscore a complex regulation of inflammatory mediators during CMV infection, potentially mediating both harmful and beneficial effects on the host. In the VICTOR study, patients were included based on local CMV viremia assessments. However, when repeating this analysis at the central laboratory with the Amplicor assay, 54 of the 289 patients with available plasma samples for the present analysis had CMV DNAemia below LLoQ. This allowed us to compare solid organ transplant recipients suspected to have CMV disease, with and without detectable CMV DNAemia. In multivariate analyses, we found a significant association between high levels of IP-10 and CRP and CMV DNAemia above LLoQ. While the association with CRP, as a reliable marker of inflammation, confirms the close interaction between inflammation and CMV replication, the association with IP-10 may be of particular interest. IP-10 has been demonstrated to be critical in providing host defense against viral infection in various organ compartments (e.g. central nervous system) [14]. Apart from its antiviral [14] and angiostatic [15] properties, IP-10 is also of major importance for the recruitment of the inflammatory type 1 T helper cells into sites of inflammation [16]. Studies on murine CMV infections have shown that IP-10 promotes the accumulation of CMV specific CD8^+^ cells in the infected liver [17]. In human microglial cells, CMV has been reported to induce IP-10 release, potentially restricting CMV replication at the site of infection [14]. IP-10 has previously been related to various viral infections such as infections caused by influenza, rhinovirus, hepatitis C and human immunodeficiency virus [18]–[20]. Although the role of IP-10 in CMV infection at present is not clear, our findings suggest a link between this chemokine and the presence of active CMV infection in solid organ transplant recipients.

While a balanced immune response against viral infections may be beneficial for the host, a persistent and imbalanced response could contribute to tissue damage and disease progression. In the present study, we found that certain inflammatory pathways, other than IP-10, were associated with disease manifestations in CMV infected transplant recipients. Thus, while there was no association between CMV viral load and tissue invasive CMV disease, high levels of PTX3 and CCL21 were independently associated with this clinical manifestation. This may suggest that tissue invasive CMV disease is not merely caused by increased CMV replication, but also involve the contribution of maladaptive immune responses.

PTX3 belongs to pentraxin protein family that also includes CRP. While CRP is produced in the liver, PTX3 is widely expressed under inflammatory stimuli including expression in neutrophils, endothelial cells, epithelial cells, macrophages and dendritic cells (reviewed in [21]). Whereas PTX3 has been reported to possess anti-viral and anti-microbial properties [22], [23], it is tempting to hypothesize that its relation to tissue invasive CMV disease reflects its ability to mirror important inflammatory responses at the site of inflammation and tissue damage during CMV related disease even better than the more “famous” pentraxin protein CRP. As for the association between CCL21 and clinical manifestation of CMV disease, several possibilities exist. CCR7, the receptor for the homeostatic chemokines CCL19 and CCL21, has been shown to be involved in the T cell specific response to CMV infection [24]. However, the association between high CCL21 levels and tissue invasive CMV disease may also reflect other CCL21-related mechanisms. Thus, whereas CCL21 plays a pivotal role in T cell and dendritic cell trafficking into lymphoid tissue, new data revealed the involvement of this chemokine also in inflammatory responses and T cell homing into non-lymphoid tissue [25]. Hence, high CCL21 levels have been related to various inflammatory disorders like inflammatory bowel disease [26], rheumatoid arthritis [27] and atherosclerosis [28]. It is possible that similar mechanisms could be operating in tissue invasive CMV disease, i.e., CCL21 could promote lymphocyte infiltration and activation at the site of CMV replication. Whatever the mechanism, our findings suggest a link between clinical manifestation of CMV disease and PTX and CCL21 levels.

Our present study suggests an association between certain inflammatory markers and tissue invasive CMV disease. While each marker in itself has a relatively low OR, our data suggest that the combined use of several markers, including the inflammatory parameters, could be of potential interest. However, the usefulness of the tested inflammatory markers in clinical practice is rather unclear and must be tested in larger study populations. Moreover, while we by no means want to imply that the tested inflammatory markers at present should replace more established diagnostic tools, our findings suggest that the identified mediators could be of potential interest in the pathogenesis of these complex disorders (i.e., tissue invasive CMV disease).

The strengths of the present study are a large patient population and the examination of a wide range of inflammatory markers simultaneously. We have also, to the best of our knowledge, identified some inflammatory mediators that not previously have been linked to clinical CMV infection in solid organ transplant recipients (e.g., IP-10 and PTX3). However, the study also has limitations. Associations do not necessarily mean any causal relationship, and the lack of mechanistic data is a limitation of the present study. There were also relatively few patients with negative viral load. Although our findings suggest that the identified mediators could be of interest in the pathogenesis of CMV related disease, future research should explore this hypothesis in more mechanistic studies that also include experimental models of CMV disease. Such studies should further explore the complex immunopathogenic mechanisms in CMV infection contributing to host defense and viral replication as well as CMV-related disease manifestations.
